# Exploratory disproportionality analysis of potentially drug-induced eosinophilic pneumonia using United States Food and Drug Administration adverse event reporting system

**DOI:** 10.1038/s41598-025-85681-0

**Published:** 2025-01-09

**Authors:** Ashwin Kamath

**Affiliations:** Department of Pharmacology, Kasturba Medical College Mangalore, Manipal Academy of Higher Education, Manipal, Karnataka India

**Keywords:** Eosinophilic pneumonia, Adverse drug reaction, Eosinophils, Early diagnosis, Antimicrobial agents, Respiratory tract diseases, Drug safety, Pharmacology

## Abstract

Drug-induced eosinophilic pneumonia (EP) is an uncommon adverse drug reaction. Many drugs have been reported to cause EP, the evidence mainly being in the form of case reports/case series. This study aims to conduct an exploratory analysis of the United States Food and Drug Administration adverse event reporting system (FAERS) database to identify previously unknown drugs that can cause EP and supplement the available evidence for known culprit drugs. A retrospective case–noncase study was conducted using individual case safety reports (ICSRs) reported to the US FAERS from the first quarter of 2004 to the second quarter of 2024. Cases of potentially drug-induced EP were identified using OpenVigil application by conducting a narrow and broad scope search using the Medical Dictionary of Regulatory Activities preferred terms. A base list of drugs described in select literature to have caused EP was used to categorize known and unknown drugs. A disproportionality analysis was performed, with a reporting odds ratio > 2, lower end of the 95% confidence interval > 1, and a minimum of 3 reported cases considered a signal of disproportionate reporting (SDR). During the study period, 8,702,548 individual case safety reports (ICSRs) were submitted to the FAERS. Of these, 855 ICSRs using the narrow scope search and 1411 ICSRs using the broad scope search reported EP. The three most commonly reported drugs with an SDR for EP using the narrow scope search were daptomycin, naltrexone, and prednisone. The most common indications for the use of the drugs were infections, immunological conditions, asthma, and central nervous system disorders. In total, there were 45 drugs with an SDR but no supporting literature evidence available. The number of drugs implicated in causing EP has increased over the years. Several antimicrobial agents, followed by drugs affecting the central nervous system and anticancer drugs, including monoclonal antibodies, can produce EP. The list of suspected drugs identified in this study, especially those with SDR and literature evidence, should be strongly considered as a possible cause in patients presenting with pneumonia not explained otherwise.

**Supplementary Information:**

The online version contains supplementary material available at 10.1038/s41598-025-85681-0.

## Introduction

Eosinophilic pneumonia is a group of disorders characterized by eosinophilic infiltration of the lung parenchyma with or without an increase in the eosinophil count in the peripheral circulation^1^. It may present as an acute or a chronic disease, as a simple respiratory illness or as acute respiratory distress syndrome, with varying etiologies, drugs being an important inciting factor^2^. Drug-induced eosinophilic pneumonia is potentially preventable and easily treated once recognized; at the same time, due to its rarity, the diagnosis is often delayed, leading to unnecessary investigations and antimicrobial administration. Several drugs, such as daptomycin, minocycline, venlafaxine, ranitidine, and paracetamol, are known to cause eosinophilic pneumonia^3^; however, due to the rarity of the event, the evidence is mainly in the form of case reports/case series. Given this, it is possible that there are drugs that can potentially cause eosinophilic pneumonia but are not yet identified or described in the scientific literature.

Adverse event databases that contain data on adverse drug reactions spontaneously reported by consumers and health professionals are excellent data sources for understanding drug-related safety issues^4,5^. Although such spontaneously reported data have many limitations, such as incomplete information, reporting bias, inaccurate data, and underreporting, the large number of reports available help in the provisional identification of potential drug safety issues^6^. Reports of drugs causing eosinophilic pneumonia, which hitherto have not been reported in the literature, may be available in these spontaneous reporting databases. Although the availability of such reports does not indicate causality, statistical analysis of the reports employing disproportionality methods has been used to identify signals of disproportionate reporting, which may warrant further clinical exploration to understand the role of the drug in causing the event. Accordingly, this study aimed to conduct an exploratory analysis of the United States FDA adverse event reporting system (FAERS) to identify previously unknown drugs that can cause eosinophilic pneumonia. FAERS was chosen because the database contributes to approximately 45% of the data in the global pharmacovigilance database VigiBase and allows free public access to the data^7^.

## Methods

A retrospective case–noncase study was conducted using individual case safety reports (ICSRs) reported to the US FAERS from the first quarter of 2004 to the second quarter of 2024. The FAERS data were accessed using the OpenVigil (version 2.1) software application^8^. Briefly, OpenVigil provides an intuitive custom user interface for drug/ADR searches of FAERS ICSRs; in addition, OpenVigil can provide disproportionality statistics, be used to identify and eliminate confounding factors, and determine the presence of potential drug-drug interactions. In the present study, OpenVigil was used to identify drugs reported to cause eosinophilic pneumonia, and drugs with signals of disproportionate reporting were identified from this list of drugs. Cases of potentially drug-induced eosinophilic pneumonia were identified by searching for the following Medical Dictionary of Regulatory Activities (MedDRA) preferred terms (PTs): eosinophilic pneumonia, acute eosinophilic pneumonia, and chronic eosinophilic pneumonia constituted a narrow scope search; eosinophilic bronchitis, eosinophilic granulomatosis with polyangiitis, eosinophilic pleural effusion, hypereosinophilic syndrome, Loeffler’s syndrome, and pulmonary eosinophilia, in addition to narrow scope PTs, constituted a broad scope search. The latter search strategy was used to identify reports wherein there may have been involvement of lung parenchyma/pleura with increased eosinophil count as part of other eosinophilic disorders, such as drug reaction with eosinophilia and systemic symptoms (DRESS) and Churg-Strauss syndrome. The PTs were selected from those constituting the standardized MedDRA query (SMQ) “eosinophilic pneumonia”^9^. ICSRs involving all ages, sexes, indications, outcomes, and reporter types, with the country of occurrence being the United States, were included in the analysis. Although the FAERS receives reports from other countries, the inclusion of such reports can confound the analysis, as such reporting is performed mostly by pharmaceutical companies for regulatory requirements (expedited and non-expedited reports); hence, reports from other countries were excluded^10,11^. Analysis was conducted for all drugs, irrespective of whether they were listed as primary or secondary suspect, concomitant or interacting drugs.

The study was approved by Institutional Ethics Committee, Kasturba Medical College, Mangalore (IEC KMC MLR 04/2024/225). Informed consent was waived due to the retrospective nature of the study and the use of publicly accessible deidentified data.

### Disproportionality analysis

To determine whether the observed reporting rate for the event was greater than that expected for a particular drug, disproportionality analysis was performed using the reporting odds ratio (ROR). The calculation of the statistic is based on a two-by-two contingency table, with the ROR being akin to the odds ratio^12^. Each case report can contribute to only one of the cells of the contingency table. Only the most recent versions of a case were included in the analysis. A ROR > 2 with a lower end of the 95% confidence interval > 1 and a minimum of 3 reported cases was considered a signal of disproportionate reporting (SDR).

### Literature search

A list of drugs (base list) reported to cause eosinophilic pneumonia was prepared, primarily based on the review of drug-induced eosinophilic pneumonia by Bartal et al., supplemented with information from the UpToDate® online drug information solution (see Additional file 1)^3,13^. This list was compared with that obtained following disproportionality analysis using a narrow and broad scope search. For drugs with SDR not present in the base list, a literature search was performed in PubMed and Google search engines using the following search string: *drug name* AND *(“eosinophilic bronchitis” OR “eosinophilic granulomatosis with polyangiitis” OR “eosinophilic pleural effusion” OR “eosinophilic pneumonia” OR “eosinophilic pneumonia acute” OR “eosinophilic pneumonia chronic” OR “hypereosinophilic syndrome” OR “Loeffler’s syndrome” OR “Loeffler syndrome” OR “pulmonary eosinophilia”)*. The presence of at least a single case report was necessary to consider a drug with SDR to be a potential etiologic factor. The absence of even a single case report for any drug with SDR was further confirmed by searching for the Pneumotox application, which is a continuously updated source of drug-induced and iatrogenic respiratory disease^14^.

## Results

From the first quarter of 2004 to the second quarter of 2024, 8,702,548 ISCRs were submitted to the FAERS.

## Eosinophilic pneumonia—narrow scope search

A total of 855 ICSRs reporting eosinophilic pneumonia were available. There were 108 unique primary suspect drugs, 17 secondary suspects, and 155 concomitant medications (see Additional file 2). The three most common drugs contributing to eosinophilic pneumonia were daptomycin with 375 cases, naltrexone 41 cases, and prednisone 39 cases. On disproportionality analysis, 72 medications showed SDR for eosinophilic pneumonia (Table 1). Twenty-one of these drugs were already reported in the base list to cause eosinophilic pneumonia based on one or more case reports. Among the rest, 30 had at least one case report available after conducting a literature search (Table 2) whereas no supporting evidence was available for 21 drugs (Table 3).

**Table 1:** List of suspect drugs for eosinophilic pneumonia (narrow scope search) with signal of disproportionate reporting.

Suspect drug	ROR	ROR		Number of AEs of interest (*n* = 855)	Number of other AEs (*n* = 8,702,548)
		Lower bound	Upper bound		
Ado-Trastuzumab emtansine	21.73	6.99	67.58	3	1410
Alemtuzumab#	6.59	2.12	20.48	3	4648
Amiodarone*	23.56	16.79	33.06	35	15,734
Amoxicillin	6.07	3.58	10.30	14	23,789
Ascorbic Acid#	14.62	4.70	45.46	3	2095
Azithromycin*	7.43	4.29	12.85	13	18,053
Aztreonam	4.40	1.65	11.76	4	9284
Balsalazide*	42.43	13.63	132.14	3	722
Benralizumab#	4.88	1.57	15.16	3	6277
Benzoyl peroxide#	19.42	7.26	51.90	4	2106
Bortezomib#	6.08	3.44	10.76	12	20,313
Bosutinib#	6.61	2.13	20.53	3	4635
Candesartan#	8.35	2.69	25.96	3	3667
Capecitabine	3.62	2.05	6.39	12	34,130
Carfilzomib	5.21	1.95	13.92	4	7844
Cefepime	32.74	18.50	57.93	12	3782
Ceftaroline fosamil*	99.15	31.74	309.74	3	309
Ceftriaxone	38.65	25.29	59.07	22	5942
Ciprofloxacin	3.49	1.81	6.73	9	26,469
Clarithromycin*	7.41	2.77	19.79	4	5519
Clavulanic Acid	11.88	5.64	25.00	7	6043
Clindamycin	3.85	1.60	9.27	5	13,285
Clopidogrel	2.79	1.64	4.73	14	51,688
Daptomycin*	1923.42	1673.73	2210.36	375	3533
Daratumumab#	6.06	2.27	16.20	4	6740
Docusate#	2.81	1.17	6.78	5	18,153
Donepezil#	11.14	5.97	20.78	10	9236
Doxycycline	8.79	5.36	14.42	16	18,839
Entacapone#	16.22	6.07	43.35	4	2521
Ertapenem#	75.95	41.81	137.97	11	1493
Ethambutol*	15.73	5.06	48.90	3	1948
Exemestane#	5.44	1.75	16.91	3	5628
Finasteride#	9.90	5.94	16.49	15	15,676
Flecainide	17.63	8.37	37.11	7	4073
Heparin	3.52	1.58	7.85	6	17,455
Hydroxychloroquine	4.20	2.37	7.42	12	29,414
Imipenem*	28.77	9.24	89.51	3	1065
Isoniazid*	18.48	6.91	49.39	4	2213
Leflunomide	13.31	8.44	20.98	19	14,836
Levofloxacin	11.55	8.19	16.27	34	31,100
Linezolid	19.69	10.20	38.00	9	4699
Meropenem	47.93	25.66	89.54	10	2148
Mesalazine*	12.67	7.60	21.11	15	12,249
Methotrexate*	2.24	1.52	3.31	26	120,105
Methylprednisolone#	8.47	5.16	13.89	16	19,551
Metronidazole	4.54	2.03	10.14	6	13,523
Minocycline*	65.34	46.32	92.17	34	5512
Montelukast	4.11	2.57	6.55	18	45,332
Naltrexone	22.46	16.41	30.75	41	19,468
Naproxen*	4.13	2.75	6.19	24	60,490
Oxaliplatin*	4.64	1.74	12.38	4	8816
Pertuzumab#	7.97	2.57	24.78	3	3842
Phenytoin*	6.89	3.69	12.85	10	14,927
Piperacillin*	24.08	13.27	43.67	11	4708
Pomalidomide	2.39	1.28	4.45	10	42,972
Prednisone#	3.69	2.68	5.09	39	111,221
Progesterone*	25.87	15.52	43.14	15	6002
Rifabutin	40.15	12.90	125.03	3	763
Rifampin	40.29	22.77	71.32	12	3073
Risperidone*	2.38	1.35	4.22	12	51,656
Selegiline#	18.73	6.02	58.24	3	1636
Sertraline*	4.60	3.14	6.75	27	61,215
Sulfamethoxazole	8.68	5.74	13.14	23	27,616
Sulfasalazine*	4.88	2.03	11.76	5	10,474
Tadalafil#	3.20	1.92	5.34	15	48,260
Tamsulosin#	5.15	3.14	8.44	16	32,116
Tazarotene#	61.87	23.10	165.73	4	661
Tazobactam	23.14	12.76	41.97	11	4899
Trastuzumab	3.43	1.28	9.16	4	11,909
Trimethoprim	8.72	5.76	13.19	23	27,513
Ustekinumab*	3.05	1.64	5.70	10	33,592
Vancomycin	19.42	13.41	28.13	29	15,703

ROR, reporting odds ratio; AE, adverse event.

*Known to have caused eosinophilic pneumonia based on literature (base list).

#No literature evidence.

**Table 2:** Drugs with signal of disproportionate reporting for eosinophilic pneumonia and supporting literature evidence.

Suspect drug	References	Pulmonary/pleural involvement described
Ado-trastuzumab emtansine	^20^	Yes
Amoxicillin	^21–24^	Yes
Aztreonam	^25^	No
Capecitabine	^26^	Yes
Carfilzomib	^27^	Yes
Cefepime	^28^	Yes
Ceftriaxone	^29,30^	No
Ciprofloxacin	^31,32^	Yes
Clavulanic acid	^33^	Yes
Clindamycin	^34,35^	Yes
Clopidogrel	^36,37^	Yes
Doxycycline	^38,39^	Yes
Flecainide	^40,41^	Yes
Heparin	^42,43^	No
Hydroxychloroquine	^44^	Yes
Leflunomide	^45,46^	Yes
Levofloxacin	^18,47^	Yes
Linezolid	^48,49^	No
Meropenem	^50–53^	Yes
Metronidazole	^54,55^	Yes
Montelukast	^56–59^	Yes
Naltrexone	^60,61^	Yes
Pomalidomide	^62,63^	Yes
Rifabutin	^25^	No
Rifampin	^64–66^	Yes
Sulfamethoxazole	^67,68^	Yes
Tazobactam	^69^	Yes
Trimethoprim	^68^	Yes
Vancomycin	^70,71^	Yes
Trastuzumab	^72,73^	Yes

**Table 3:** Drugs showing signal of disproportionate reporting of eosinophilic pneumonia but without supporting literature.

Based on narrow scope search	Based on broad scope search
Alemtuzumab	Beclomethasone
Ascorbic acid	Benzatropine
Benralizumab	Budesonide
Benzoyl peroxide	Docetaxel
Bortezomib	Fluticasone
Bosutinib	Formoterol
Candesartan	Magnesium sulfate
Daratumumab	Memantine
Docusate	Mepolizumab
Donepezil	Metronidazole
Entacapone	Mometasone
Ertapenem	Prednisolone
Exemestane	Salmeterol
Finasteride	Theophylline
Methylprednisolone	
Pertuzumab	
Prednisone	
Selegiline	
Tadalafil	
Tamsulosin	
Tazarotene	

**Fig. 1:**
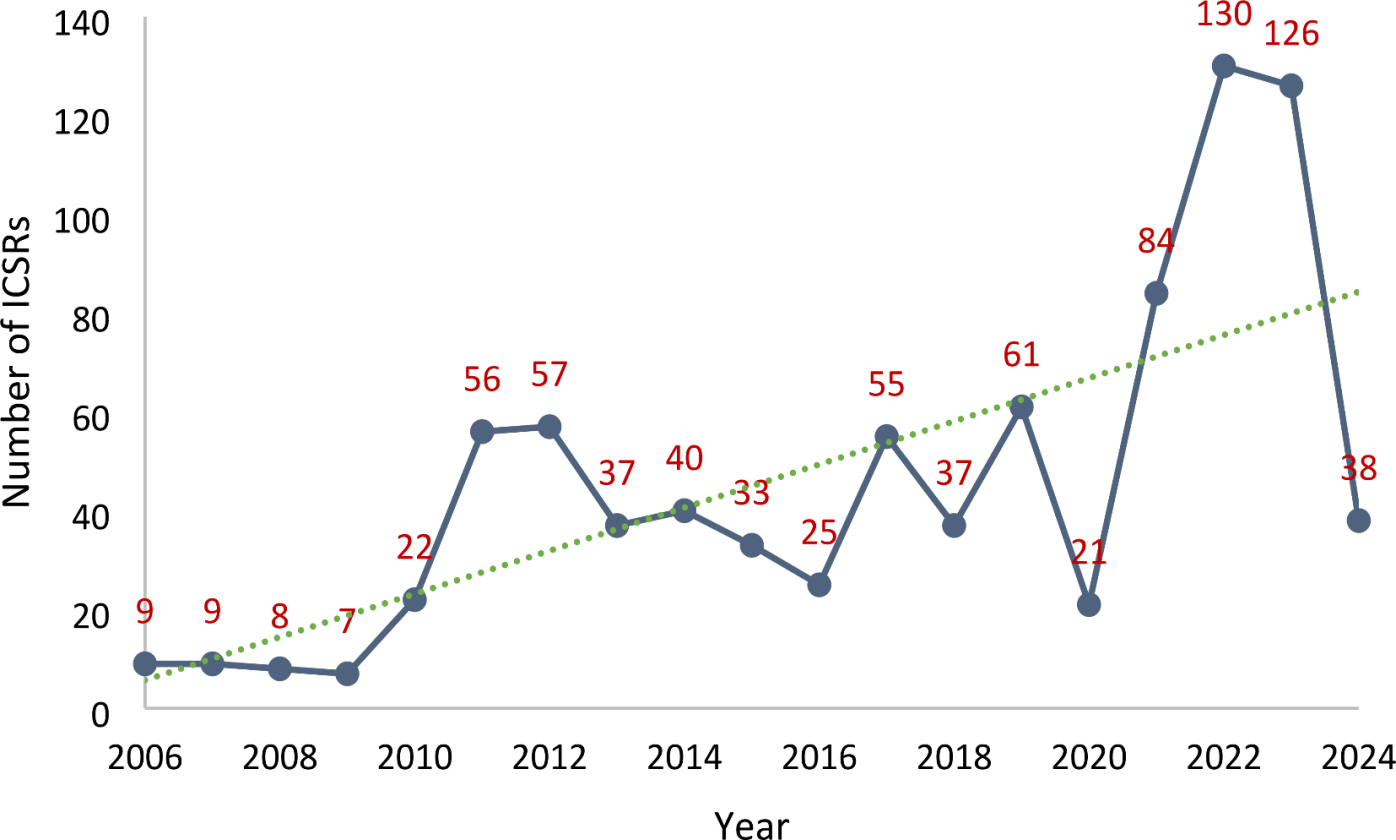
Number of ICSRs of eosinophilic pneumonia (narrow scope search) reported yearly to United States FAERS. The green dotted line indicates the reporting trend over the years. ICSR, individual case safety report; FAERS, Food and Drug Administration adverse event reporting system.

The annual reporting of ICSRs of eosinophilic pneumonia to the FAERS is shown in Fig. 1. Regarding gender, 422 were males, 253 females, and gender was not reported in 180 ICSRs. The mean ages (mean ± standard deviation) were 62.77 ± 16.85 years and 51.23 ± 21.14 years for males and females, respectively. Indications for drug use were mentioned in 556 ICSRs; the most common indications were osteomyelitis (50/556 [8.99%]); staphylococcal infection (24/556 [4.32%]); alcoholism (17/556 [3.06%], each), ulcerative colitis, rheumatoid arthritis (15/556 [2.70%], each); depression (13/556 [2.34%]); plasma cell myeloma, schizophrenia (12/556 [2.16%], each); asthma, and atrial fibrillation (10/556 [1.79%], each). Of the 855 cases, 37 (4.33%) died, 381 (44.56%) required hospitalization or prolongation of hospitalization, and 90 (10.53%) had a life-threatening reaction with or without other serious outcomes.

### Eosinophilic pneumonia—broad scope search

Of all the ICSRs, 1411 reported eosinophilic pneumonia as an adverse event. There were 175 unique primary suspect drugs, 32 secondary suspects, and 174 concomitant medications. On disproportionality analysis using the broad scope, 93 medications showed SDR for eosinophilic pneumonia; of these, 64 also had SDR for eosinophilic pneumonia using the narrow search scope; the remaining 29 medications are listed in Table 4. Of these, 5 drugs were already mentioned in the base list. Among the rest, 12 had at least one case report available after conducting a literature search (Table 5) whereas no supporting evidence was available for 12 drugs (Table 3).

**Table 4:** List of suspect drugs for eosinophilic pneumonia (broad scope search) with signal of disproportionate reporting.

Suspect drug	ROR	ROR		Number of AEs of interest (*n* = 1411)	Number of other AEs (*n* = 8,702,548)
		Lower bound	Upper bound		
Allopurinol	2.15	1.15	4.00	10	28,816
Amitriptyline	5.13	3.23	8.18	18	21,843
Azathioprine	4.82	2.41	9.67	8	10,276
Beclomethasone#	5.84	1.88	18.13	3	3175
Benzatropine#	15.56	9.18	26.35	14	5602
Biotin	3.58	1.34	9.56	4	6899
Budesonide#	2.09	1.31	3.33	18	53,401
Cilastatin*	28.05	9.01	87.29	3	661
Clozapine*	2.97	1.89	4.66	19	39,869
Docetaxel#	2.66	1.47	4.82	11	25,596
Dupilumab	2.09	1.63	2.69	64	193,288
Fluticasone#	2.59	1.85	3.65	34	82,038
Imatinib	3.39	1.69	6.78	8	14,634
Levetiracetam	2.63	1.55	4.46	14	33,010
Memantine#	7.98	4.14	15.38	9	6990
Mepolizumab#	21.52	14.98	30.92	30	8774
Methimazole	12.84	5.76	28.66	6	2892
Mirtazapine	4.07	2.25	7.37	11	16,756
Mometasone#	2.77	1.44	5.33	9	20,149
Nitrofurantoin*	3.71	1.39	9.91	4	6658
Omalizumab	7.41	5.28	10.42	34	28,881
Paliperidone	2.13	1.14	3.97	10	29,056
Prednisolone#	5.41	2.99	9.79	11	12,623
Salbutamol#	2.59	1.96	3.41	52	126,918
Salmeterol#	4.16	2.96	5.84	34	51,373
Theophylline#	7.77	2.50	24.12	3	2387
Topiramate	2.12	1.17	3.83	11	32,166
Valproic acid*	9.15	6.37	13.15	30	20,602
Zafirlukast	29.66	11.09	79.32	4	834

ROR, reporting odds ratio; AE, adverse event.

*Known to have caused eosinophilic pneumonia based on literature.

#No literature evidence.

**Table 5:** Drugs with signal of disproportionate reporting for eosinophilic pneumonia (broad scope search) and supporting literature evidence.

Suspect drug	References	Pulmonary/pleural involvement described
Allopurinol	^74,75^	Yes
Amitriptyline	^76^	Yes
Azathioprine	^77,78^	Yes
Biotin	^79,80^	Yes
Clozapine	^81–83^	Yes
Dupilumab	^84–87^	Yes
Imatinib	^88–90^	Yes
Methimazole	^91^	Yes
Mirtazapine	^92,93^	No
Omalizumab	^94–97^	Yes
Paliperidone	^98^	Yes
Topiramate	^66^	No
Zafirlukast	^99–101^	Yes

**Fig. 2:**
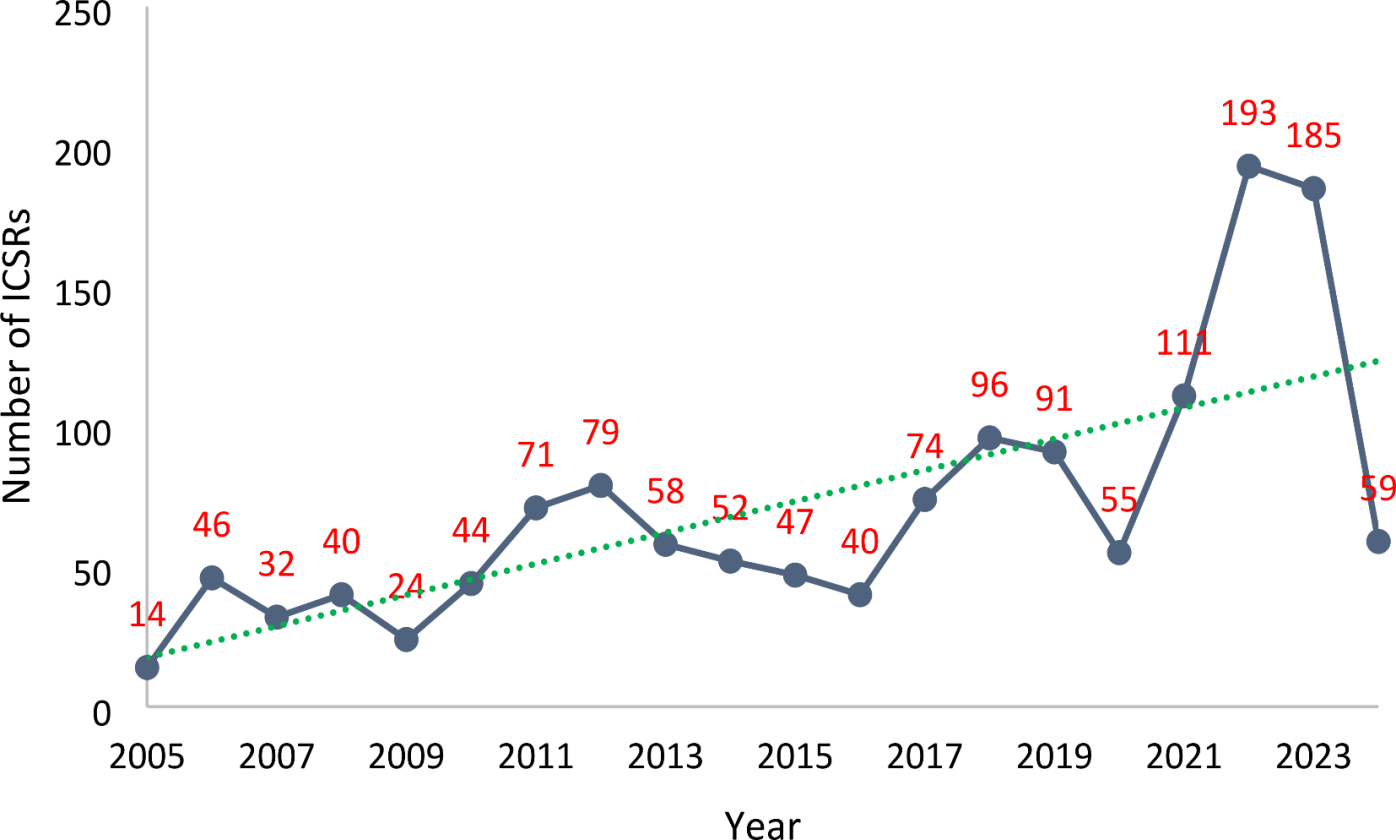
Number of ICSRs of eosinophilic pneumonia (broad scope search) reported yearly to United States FAERS. The green dotted line indicates the reporting trend over the years. ICSR, individual case safety report; FAERS, Food and Drug Administration adverse event reporting system.

The annual reporting of ICSRs of eosinophilic pneumonia to the FAERS is shown in Fig. 2. Regarding gender, 621 were males, 507 females, and gender was not reported in 283 ICSRs. The mean ages were 58.61 ± 18.58 years and 51.65 ± 19.98 years for males and females, respectively. Indications for drug use were mentioned in 961 ICSRs; the most common indications were asthma (124/961 [12.90%]); osteomyelitis (51/961 [5.31%]); rheumatoid arthritis (27/961 [2.81%]); ulcerative colitis (26/961 [2.71%]); staphylococcal infection (24/961 [2.50%]); and schizophrenia (23/961 [2.39%]). Of the 1411 cases, 64 (4.54%) died, 594 (42.10%) required hospitalization or prolongation of hospitalization, and 141 (9.99%) had a life-threatening reaction with or without other serious outcomes.

Combining both narrow and broad scope searches, all the drugs with SDR were listed as primary or secondary suspect drugs in one or more ICSRs except for the following drugs, which were listed only as concomitant medications in cases with eosinophilic pneumonia: ascorbic acid, benzatropine, benzoyl peroxide, biotin, docusate, ethambutol, exemestane, finasteride, flecainide, hydroxychloroquine, memantine, mirtazapine, rifabutin, tadalafil, tamsulosin, tazarotene, and theophylline. There were 45 drugs mentioned in the base list that did not show an SDR on disproportionality analysis (see Additional file 1).

## Discussion

Drug-induced eosinophilic pneumonia is a relatively rare adverse effect characterized by elevated blood eosinophil counts, eosinophilic infiltration of the lungs, and increased eosinophils in the bronchoalveolar lavage fluid^1^. It is often initially mistaken for infective pneumonia and hence is easily missed. The importance of early detection lies in the fact that the event generally responds well to corticosteroid therapy and, in the absence of appropriate treatment, can result in serious outcomes, in addition to the consequences of unnecessary antibiotic therapy. Although several drugs have been reported to cause the adverse reaction, the evidence is mostly from case reports/series. In the present study, extensive data from the FAERS database was leveraged to identify drugs that disproportionately reported eosinophilic pneumonia. A literature search was conducted to determine whether there were any published data to support the identified SDR.

The mean age of the patients with potentially drug-induced eosinophilic pneumonia in this study was just above 50 years, with an increased prevalence in males. Earlier studies have described a common age range of 20–40 years and a bipeak prevalence at 15–24 years and 65–79 years^15^; however, these studies included eosinophilic pneumonia due to all causes. Moreover, the FAERS data is limited by underreporting and a lack of data capture in all ICSRs, and the clinical studies are limited by small sample sizes and varying geography. In general, age and sex do not seem to be predominant factors that determine the occurrence of the event. The common clinical indications for which the suspected drugs were administered include infections, particularly osteomyelitis and staphylococcal infection, CNS disorders, asthma, and immunological diseases. This is in line with the suspected drugs contributing to the highest number of cases, including daptomycin, montelukast, prednisone, levofloxacin, and naltrexone. Additionally, most of the drugs with SDR are antimicrobial drugs, followed by drugs for CNS disorders, including psychotropic drugs, and equal contributions from antiasthmatic drugs, anticancer drugs, and endocrine drugs. In particular, daptomycin accounted for 43.94% of the ICSRs in the narrow-scope search and 27.30% of the ICSRs in the broad-scope search, with the contributions of all other drugs being significantly less, except for montelukast (14.30%) in the broad-scope search.

A total of 62 drugs showed an SDR for eosinophilic pneumonia; upon broadening the search to include other eosinophilic disorders, 30 additional drugs with SDR were identified. Taken together, 24 drugs were already present in the base list; thus, the literature evidence for these drugs is further strengthened by the presence of SDR. Thirty-nine drugs with SDR, not present in the base list, had at least one supporting case report associating them with eosinophilic disorder. These drugs should be considered in the etiology of drug-induced eosinophilic pneumonia. Daptomycin is the most common culprit drug according to FAERS analysis and the literature. The exact mechanism is unknown. Direct action in the form of epithelial injury and alteration of surfactant function or indirect effects by stimulating alveolar macrophages and inducing interleukin 5 release from T-helper 2 lymphocytes to promote pulmonary accumulation of eosinophils have been proposed as potential mechanisms^16^. Whether similar mechanisms are involved in the response to other drugs is not known. The FDA has defined a case of potentially daptomycin-induced eosinophilic pneumonia to satisfy the following criteria: concurrent exposure to the drug, fever, dyspnea with increased oxygen requirement or requiring mechanical ventilation, new infiltrates on chest X-ray or computed tomography scan, bronchoalveolar lavage with > 25% eosinophils, and clinical improvement following withdrawal of the drug^17^. Of particular interest are levofloxacin and naltrexone, which were not present in the base list but contributed to > 25 ICSRs, both in the narrow and broad scope of the search. For the former, eosinophilic pneumonia due to the use of levofloxacin eye drops has been reported; the reaction has been attributed to delayed allergy due to cellular immunity^18^.

There were 29 drugs that showed an SDR, but there was no supporting published literature. Notably, many drugs used to treat eosinophilic disorders, such as alemtuzumab, mepolizumab, formoterol, and glucocorticoids, have shown an SDR in disproportionality analysis for eosinophilic pneumonia. These cases are likely a result of confounding by indication. Another potential reason could be a decrease in the dose or withdrawal of steroids in these patients, which can aggravate a pre-existing eosinophilic disorder, as has been observed in the case of dupilumab^19^. In the case of some drugs, like heparin and leucovorin, the literature evidence is rather weak. For heparin, only eosinophilia is described but not pulmonary involvement. Regarding leucovorin case reports, the drug has always been administered with other anticancer drugs, and hence, the event cannot be attributed specifically to leucovorin. Although there is no literature evidence for some drugs, such as alemtuzumab, pertuzumab, ertapenem, and candesartan, other related drugs are known to cause eosinophilic pneumonia; hence, the SDR observed for these drugs merits further study in the form of active surveillance.

Conversely, there were 45 drugs in the base list that were described in the literature to cause eosinophilic pneumonia but did not show statistically significant results on disproportionality analysis. These contradictory findings are not completely unexpected given that the FAERS data have several limitations, such as underreporting, the presence of duplicate records, and variability in reporting with time, which can result in false positive signals. However, the strength of the current study is that the SDRs were further confirmed by the presence/absence of supporting literature, which strengthens, although does not confirm, the causal relationship.

This study also has other limitations. The results from disproportionality analysis based on FAERS data are only hypothesis-generating and cannot be considered confirmatory. A literature search was employed to overcome this drawback. The literature evidence was mainly in the form of case reports/case series, which may not provide high-quality evidence. While some case reports have described the exclusion of alternative possible causes for eosinophilic pneumonia, the causality assessment was not rigorous in all the case reports. Given that one case report implicating the drug was considered adequate to label a drug as a cause for eosinophilic pneumonia, there is a possibility of false positives. However, given the rarity of drug-induced eosinophilic pneumonia, the combined approach of disproportionality analysis and a literature search provides clinically actionable evidence, albeit with limitations. Some drugs which showed SDR, such as cilastatin and clavulanic acid, are always used in combination with other drugs. However, these have been listed individually, in accordance with the results generated in OpenVigil due to the manner in which these drugs have been reported in the ICSRs. Hence, the SDR seen with these drugs cannot be attributed entirely to the drug but should be considered for the combination (i.e., imipenem and cilastatin; amoxicillin and clavulanic acid).

## Conclusions

Drug-induced eosinophilic pneumonia is an uncommon adverse effect that requires a high degree of suspicion to avoid unnecessary antimicrobial therapy and serious outcomes due to delays in stopping the offending drug and/or initiating glucocorticoid therapy. The number of drugs implicated in causing eosinophilic pneumonia has increased over the years. Apart from daptomycin, for which eosinophilic pneumonia is a well-recognized adverse effect, several other antimicrobial agents can cause this reaction, followed by drugs affecting the central nervous system and anticancer drugs, including monoclonal antibodies. The list of suspected drugs identified in this study, especially those with SDR and literature evidence, should be strongly considered as a possible cause in patients presenting with pneumonia not explained otherwise.

## Electronic supplementary material

Below is the link to the electronic supplementary material.

Supplementary Material 1

Supplementary Material 2

## Data Availability

The datasets used and/or analysed during the current study are available from the corresponding author on reasonable request.
