# Supplementary material for: Exploratory disproportionality analysis of potentially drug-induced eosinophilic pneumonia using United States Food and Drug Administration adverse event reporting system
Source: Sci Rep. 2025 Jan 9;15:1455. doi: 10.1038/s41598-025-85681-0 (PMC11718270; doi:10.1038/s41598-025-85681-0)
Supplement: Supplementary file 1 — Supplementary Material 1 [file 41598_2025_85681_MOESM1_ESM.docx]

**Additional file 1.** List of drugs reported to have caused eosinophilic pneumonia^3,13^

| **Anticancer drugs** | **Cardiovascular drugs** | **Immunomodulators** |
| --- | --- | --- |
| Cisplatin | Amiodarone* | Abatacept |
| Fludarabine | Captopril | Tacrolimus |
| Gemcitabine | Diltiazem | Infliximab |
| Methotrexate* | Mexiletine | Interferon alpha |
| Oxaliplatin* | Simvastatin | Ustekinumab* |
| Tegafur/uracil |  |  |
| **Antimicrobials** | **CNS drugs** | **NSAIDs** |
| Ampicillin | Amitriptyline*/maprotiline | Acetaminophen |
| Atovaquone/proguanil | Carbamazepine | Diclofenac |
| Azithromycin* | Clozapine* | Ibuprofen |
| Cefaclor | Duloxetine | Naproxen* |
| Ceftaroline* | Ifenprodil | Nimesulide |
| Clarithromycin* | Levetiracetam* | Piroxicam |
| Daptomycin* | Paroxetine |  |
| Dapsone/pyrimethamine | Phenytoin* | **Others** |
| Ethambutol* | Risperidone* | Aminoglutethimide |
| Imipenem/cilastatin* | Sertraline* | Benzbromarone |
| Isoniazid* | Trazodone | Bucillamine |
| Sulfadoxine/pyrimethamine | Valproic acid* | Dabigatran |
| Mefloquine | Venlafaxine | Iodides |
| Minocycline* |  | L-tryptophan |
| Nitrofurantoin* | **Gastrointestinal drugs** | Progesterone* |
| Piperacillin/tazobactam* | Balsalazide* | Sodium cromoglycate |
| Roxithromycin | Camostat mesilate |  |
| Tetracycline | Mesalamine* |  |
| Tosufloxacin | Ranitidine |  |
| Inhaled pentamidine | Sulfasalazine* |  |

*Showed signal of disproportionate reporting for eosinophilic pneumonia

The drugs have been listed under various therapeutic classes based on their predominant use.
