# Supplementary material for: Exploratory disproportionality analysis of potentially drug-induced eosinophilic pneumonia using United States Food and Drug Administration adverse event reporting system
Source: Sci Rep. 2025 Jan 9;15:1455. doi: 10.1038/s41598-025-85681-0 (PMC11718270; doi:10.1038/s41598-025-85681-0)
Supplement: Supplementary file 2 — Supplementary Material 2 [file 41598_2025_85681_MOESM2_ESM.docx]

**Additional file 2.** Drugs reported in ICSRs of eosinophilic pneumonia (narrow scope search) in United States FAERS*

| Acetaminophen | Baclofen | Calcium | Dabigatran | Eculizumab | Famotidine | Gabapentin |
| --- | --- | --- | --- | --- | --- | --- |
| Acetaminophen / Hydrocodone | Balsalazide | Candesartan | Dalfopristin / Quinupristin | Enoxaparin | Finasteride | Ganirelix |
| Acetaminophen / Oxycodone | Bendamustine | Capecitabine | Dalteparin | Entacapone | Flecainide | Gemcitabine |
| Adalimumab | Benralizumab | Carbidopa / Levodopa | Daptomycin | Epinephrine | Fluconazole | Glipizide |
| Ado-Trastuzumab emtansine | Benzatropine | Carboplatin | Daratumumab | Epoetin Alfa | Fludarabine | Guaifenesin |
| Albendazole | Benzoyl Peroxide | Carfilzomib | Darbepoetin alfa | Epoprostenol | Fluorouracil |  |
| Alemtuzumab | Benzyl Alcohol | Carvedilol | Desvenlafaxine | Ertapenem | Fluoxetine |  |
| Allopurinol | Bevacizumab | Cefazolin | Dexamethasone | Escitalopram | Fluticasone furoate / Vilanterol |  |
| Alprazolam | Bleomycin | Cefdinir | Dextroamphetamine | Esomeprazole | Fluticasone propionate |  |
| Amantadine | Bortezomib | Cefepime | Diazepam | Ethambutol | Fluticasone propionate / Salmeterol |  |
| Ambrisentan | Bosentan | Cefpodoxime | Diclofenac | Ethinyl estradiol / Levonorgestrel | Folic acid |  |
| Amiodarone | Bosutinib | Ceftaroline fosamil | Diltiazem | Ethinyl estradiol / Norethindrone | Follitropin |  |
| Amitriptyline | Botulinum Toxin Type A / Daxibotulinumtoxina | Ceftriaxone | Diphenhydramine | Etoposide | Furosemide |  |
| Amlodipine | Budesonide | Celecoxib | Divalproex | Exemestane |  |  |
| Amoxicillin / Clavulanate | Buprenorphine / Naloxone | Cephalexin | Docusate | Exenatide |  |  |
| Amphetamine | Bupropion | Cetirizine | Donepezil |  |  |  |
| Ampicillin |  | Chlordiazepoxide | Doxycycline |  |  |  |
| Anastrozole |  | Chlorthalidone | Duloxetine |  |  |  |
| Apixaban |  | Cholecalciferol | Dupilumab |  |  |  |
| Ascorbic acid |  | Choriogonadotropin alfa |  |  |  |  |
| Aspirin |  | Cilastatin / Imipenem |  |  |  |  |
| Atenolol |  | Ciprofloxacin |  |  |  |  |
| Atorvastatin |  | Ciprofloxacin / Hydrocortisone / Benzyl alcohol |  |  |  |  |
| Azacitidine |  | Citalopram |  |  |  |  |
| Azathioprine |  | Clarithromycin |  |  |  |  |
| Azelastine |  | Clindamycin |  |  |  |  |
| Azithromycin |  | Clomifene |  |  |  |  |
| Aztreonam |  | Clonazepam |  |  |  |  |
|  |  | Clonidine |  |  |  |  |
|  |  | Clopidogrel |  |  |  |  |
|  |  | Clozapine |  |  |  |  |
|  |  | Colchicine |  |  |  |  |
|  |  | Conjugated estrogens |  |  |  |  |
|  |  | Cromoglicic Acid |  |  |  |  |
|  |  | Cyanocobalamin |  |  |  |  |
|  |  | Cyclobenzaprine |  |  |  |  |
|  |  | Cyclophosphamide |  |  |  |  |
|  |  |  |  |  |  |  |
| Heparin | Ibuprofen | Lamotrigine | Magnesium sulfate | Nafcillin | Ocrelizumab | Paclitaxel |
| Hydralazine | Immune globulin human | Lansoprazole | Maribavir | Naltrexone | Omalizumab | Paliperidone |
| Hydrochlorothiazide | Infliximab | Leflunomide | Melatonin | Naltrexone / Bupropion | Omeprazole | Paliperidone |
| Hydrochlorothiazide / Losartan | Insulin | Lenalidomide | Meloxicam | Naproxen | Ondansetron | Pantoprazole |
| Hydrochlorothiazide / Triamterene | Ipilimumab | Leucovorin | Mepolizumab | Natalizumab | Oritavancin | Paroxetine |
| Hydrocortisone | Ipratropium bromide / Salbutamol | Levetiracetam | Meropenem | Nifedipine | Osimertinib | Pembrolizumab |
| Hydromorphone | Iron | Levofloxacin | Mesalazine | Nitric oxide | Oxaliplatin | Pentoxifylline |
| Hydroxychloroquine | Isoniazid | Levothyroxine | Metamizole | Nivolumab | Oxybutynin | Pertuzumab |
| Hydroxyzine | Isosorbide mononitrate | Lidocaine | Metaxalone |  |  | Phenytoin |
|  | Ivermectin | Linezolid | Metformin |  |  | Piperacillin / Tazobactam |
|  | Ixabepilone | Lisinopril | Methimazole |  |  | Pirfenidone |
|  |  | Loperamide | Methotrexate |  |  | Polyethylene glycol |
|  |  | Lorazepam | Methylprednisolone |  |  | Pomalidomide |
|  |  | Losartan | Metoprolol |  |  | Pravastatin |
|  |  | Loxoprofen | Metronidazole |  |  | Prednisolone |
|  |  | Lurasidone | Minocycline |  |  | Prednisone |
|  |  | Lutein | Mirtazapine |  |  | Pregabalin |
|  |  |  | Montelukast |  |  | Procaterol |
|  |  |  | Morphine |  |  | Progesterone |
|  |  |  | Moxifloxacin |  |  |  |
|  |  |  | Mycophenolate mofetil |  |  |  |
|  |  |  |  |  |  |  |
| Quetiapine | Rasagiline | Salbutamol | Tacrolimus | Ustekinumab | Valaciclovir | Warfarin |
|  | Ravulizumab | Salmeterol | Tadalafil |  | Valsartan |  |
|  | Remdesivir | Selegiline | Tamsulosin |  | Vancomycin | Zeaxanthin |
|  | Rifabutin | Semaglutide | Tazarotene |  | Varenicline | Zolpidem |
|  | Rifampin | Senna glycoside / Docusate / Sennosides | Telavancin |  | Vedolizumab |  |
|  | Risperidone | Sertraline | Terazosin |  | Venlafaxine |  |
|  | Rituximab | Sesame oil | Testosterone |  | Verapamil |  |
|  | Rivaroxaban | Sildenafil | Theophylline |  | Vitamin C / Cyanocobalamin / Ascorbic Acid / Riboflavin / Thiamine / Vitamin A / Pyridoxine / Cholecalciferol / Nicotinamide / Vitamin D |  |
|  | Rosuvastatin | Simvastatin | Thiamine |  |  |  |
|  |  | Sirolimus | Tigecycline |  |  |  |
|  |  | Somatropin recombinant | Tiotropium |  |  |  |
|  |  | Sotalol | Tocilizumab |  |  |  |
|  |  | Sulfamethoxazole / Trimethoprim | Topiramate |  |  |  |
|  |  | Sulfasalazine | Tramadol |  |  |  |
|  |  | Sulindac | Trastuzumab |  |  |  |
|  |  | Sumatriptan | Trazodone |  |  |  |

ICSR, individual case safety report; FAERS, Food and Drug Administration adverse event reporting system

* The listed drugs include those with or without signals of disproportionate reporting.

Red colored font indicates primary suspect drugs, brown font indicates secondary suspect drugs, and blue font indicates concomitant medications.
